# A Rare Case of Symptomatic Anomalous Origin of the Right Coronary Artery With a High Interarterial Course Between the Pulmonary Artery and the Aorta

**DOI:** 10.7759/cureus.64940

**Published:** 2024-07-19

**Authors:** Muhammad Bilal, Aamir Saeed, Ali Z Ansari, Sean Lief, Srihita Patibandla, Kotikalapudi Sivarama, Abhishek Jaiswal

**Affiliations:** 1 Department of Internal Medicine, Merit Health Wesley, Hattiesburg, USA; 2 Department of Pathology, William Carey University College of Osteopathic Medicine, Hattiesburg, USA; 3 Department of Internal Medicine, William Carey University College of Osteopathic Medicine, Hattiesburg, USA; 4 Department of Internal Medicine, Trinity Health Grand Rapids, Grand Rapids, USA; 5 Department of Interventional Cardiology, Merit Health Wesley, Hattiesburg, USA

**Keywords:** anomalous origin of the right coronary artery, coronary artery bypass grafting (cabg), congenital cardiac malformation, atherosclerotic cardiovascular disease, mechanical compression, multidetector computed tomography (mdct), transesophageal echocardiography (tee), sudden cardiac death (scd), left heart catheterization, nuclear stress test

## Abstract

The congenital anomalous origin of the right coronary artery (AORCA) with an incongruous course is a rare malformation that can manifest as exertional chest pain, syncope, arrhythmias, heart failure, and sudden cardiac death. We present a case of a 42-year-old male with a history of hypercholesterolemia who presented with chest pain and dizziness upon exertion for two weeks. The physical examination was unremarkable, and the patient was hemodynamically stable. Initial blood tests were normal. Electrocardiogram (ECG) showed sinus bradycardia at 56 bpm without ST or T wave changes. A cardiac stress test indicated antero-apical inducible ischemia with a moderate probability of stress-induced ischemia. Computed tomography angiography (CTA) revealed an AORCA with a high interarterial course between the pulmonary artery and the aorta. Subsequent left heart catheterization confirmed the anomalous origin and revealed atherosclerotic disease. This anomaly was identified as the cause of the patient’s symptoms due to the compression of the right coronary artery (RCA). The patient was treated with aspirin and statin and underwent successful internal mammary artery-RCA bypass grafting. Postoperatively, the patient’s symptoms resolved, and there were no further episodes of chest pain.

## Introduction

The congenital anomalous origin of the right coronary artery (AORCA) was first described by White and Edwards in 1948. The incidence of AORCA, particularly when arising from the left coronary cusp and coursing between the great vessels, ranges from 0.026% to 0.250% [1]. In a study of 1,960 patients, Angelini et al. found a 5.6% incidence of anomalous coronary arteries on angiography [2]. There are three recognized subtypes of anomalous RCA: a malignant high interarterial course between the pulmonary artery and the aorta, a low interarterial course between the right ventricular outflow tract and the aorta, and a hypoplastic anomalous right coronary artery (RCA) orifice. The high interarterial course is particularly rare and poses a higher risk of arrhythmias and sudden cardiac death (SCD).

Mechanical compression of the anomalous RCA during exertion or sports activities can lead to angina, myocardial infarctions, syncope, and SCD [3]. Routine testing, including resting or stress electrocardiogram (ECG), is not sensitive for detecting these congenital anomalies and does not effectively predict mortality or SCD risk in young athletes [4]. Anomalous RCA is often incidentally discovered on computed tomography angiography (CTA) in asymptomatic patients and is usually managed conservatively. However, symptomatic patients typically require surgical interventions such as unroofing or coronary artery bypass grafting.

This article was previously presented as a meeting poster at the 2023 Heart Failure Society of America (HFSA) Annual Scientific Meeting on October 8, 2023.

## Case presentation

A 42-year-old male presented to the outpatient clinic with atypical chest pain and exertional dizziness lasting for two weeks. He denied experiencing syncope, palpitations, sweating, or exertional dyspnea. His medical history included depression and hypercholesterolemia, with no significant family history of ischemic heart disease. On initial examination, his blood pressure was 126/56 mmHg, heart rate was 58 beats per minute, respiratory rate was 20 breaths per minute, and SpO2 level was 96% on room air. The physical examination and initial laboratory workup, including complete blood count, liver enzymes, and kidney function tests, were unremarkable. An ECG showed sinus bradycardia without ST-segment abnormalities.

**Figure 1:**
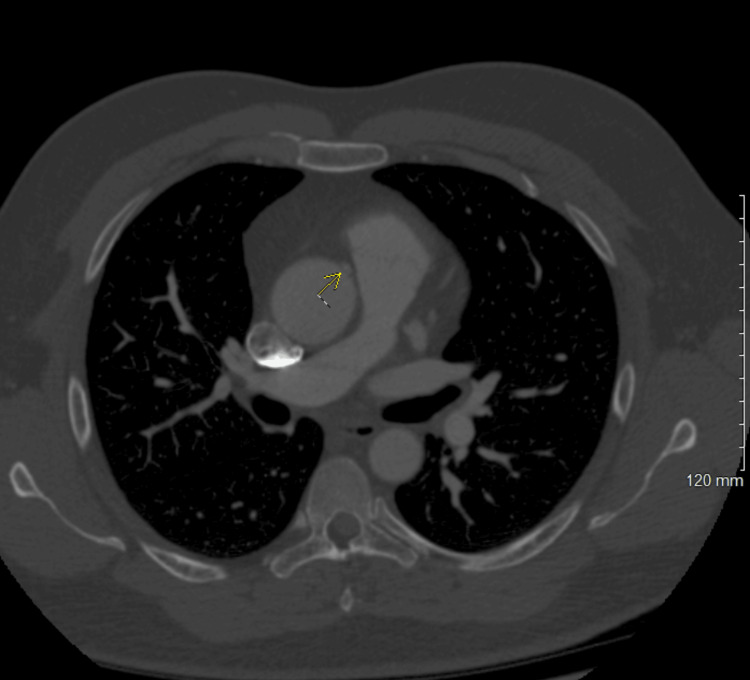
CTA displaying the AORCA (yellow arrow). CTA, computed tomography angiography; AORCA, anomalous origin of the right coronary artery

**Figure 2:**
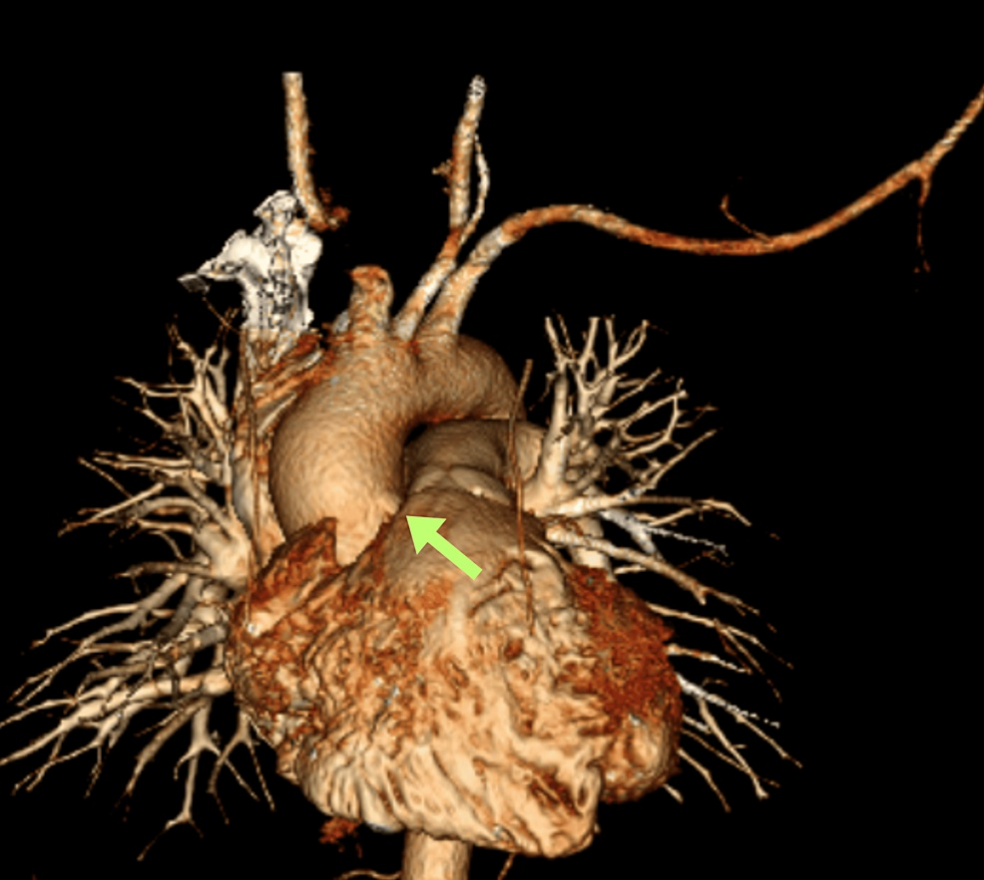
Three-dimensional reconstruction illustrating the tortuous course of the RCA (yellow arrow). RCA, right coronary artery

**Figure 3:**
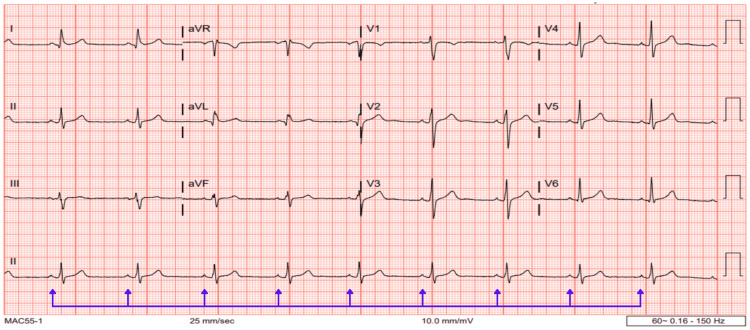
A 12-lead ECG demonstrating sinus bradycardia (blue arrows). ECG, electrocardiogram

The patient was referred to cardiology for further evaluation. A nuclear stress test indicated antero-apical ischemia with a moderate probability of stress-induced ischemia. CTA was negative for acute pulmonary embolism but revealed an AORCA with a high interarterial course between the pulmonary artery and the aorta (Figure 1). A three-dimensional reconstruction was generated to enhance visualization of the tortuous course of the RCA (Figure 2). A 6-lead ECG was ordered, which demonstrated sinus bradycardia without any ST segment or T wave changes (Figure 3).

**Figure 4:**
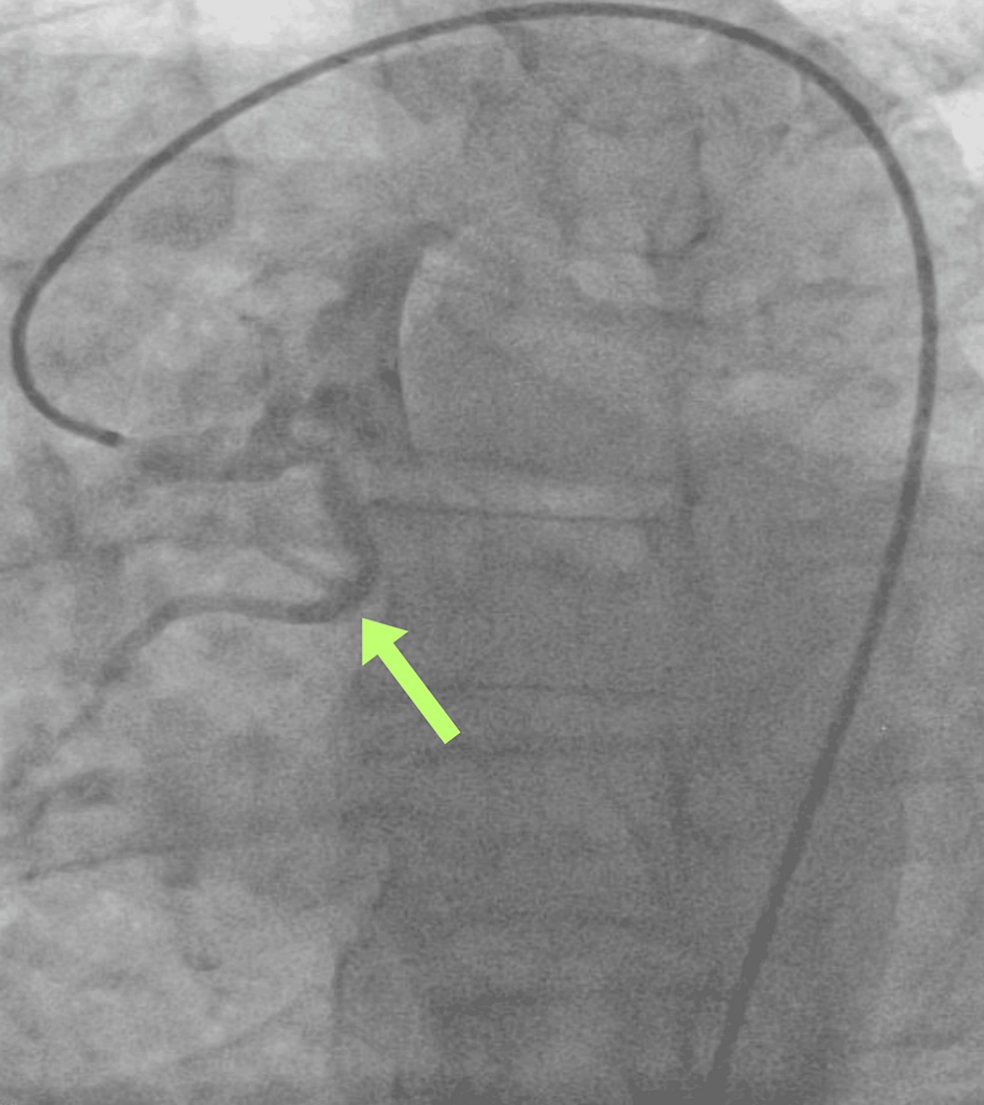
Image captured during left heart catheterization showing the anomalous course of the RCA (yellow arrow). RCA, right coronary artery

Following cardiac evaluation, the patient underwent left heart catheterization, revealing a normal left anterior descending artery (LAD) and normal circumflex artery with preserved systolic functions. Notably, an AORCA was identified, characterized by a higher interarterial course and associated atherosclerotic disease (Figure 4). Daily aspirin therapy was initiated. Cardiothoracic surgery consultation was sought, resulting in the patient undergoing coronary artery bypass grafting.

We posit that the patient's intermittent atypical chest pain and exertional dizziness upon presentation were attributable to compression of the anomalous origin of the RCA due to its malignant course between the pulmonary artery and the aorta. The procedure involved an internal mammary artery-right coronary artery (IMA-RCA) bypass, performed without complications. Subsequently, the patient was discharged one week postoperatively. At the four-week follow-up, the patient reported complete resolution of symptoms and denied experiencing any new complaints.

## Discussion

RCA arises from the right coronary sinus with a course posterior to the pulmonary artery and descending through the right atrioventricular sulcus toward the posterior interventricular septum [5]. The most common courses of congenital AORCA are high interarterial and low interarterial courses. These various courses have been associated with up to 30% of SCD. The prevalence of congenital anomalous coronary arteries is between 0.1% and 1.0% in the general population. Yamanaka and Hobbs reported the incidence to be 0.3% in more than 100,000 adults evaluated with coronary angiography [6]. Another retrospective analysis of 10,928 patients by Albuquerque et al. revealed the prevalence of AORCA with interarterial course between the pulmonary artery and the aorta was 0.26% [7].

Coronary artery anomalies have been associated with various cardiac symptoms; however, the true pathophysiology behind symptoms is poorly understood. During strenuous activities or exercise, increased cardiac output can cause pinching on the anomalous RCA due to the high course between the pulmonary artery and the aorta [8]. This dynamic narrowing and kinking can lead to myocardial infarctions, ventricular arrhythmias, and SCD. Others have suggested that the pathophysiology behind the symptoms is the oblique angle between anomalous RCA and left coronary sinus that can produce slit-like orifice, abnormal takeoff of the RCA origin, or propensity of the spasm of the proximal segment [9]. Coronary anomalies are associated with up to one-third of SCDs in the young population, especially with an intramural and interarterial course; therefore, early diagnosis of such anomalies is crucial [10].

There are several methods to evaluate AORCA, including echocardiography, magnetic resonance imaging (MRI), multidetector computed tomography (MDCT), angiography, and transesophageal echocardiography (TEE). Compared with invasive procedures, MDCT allows more accurate information about the origin and course of the anomalous artery. MDCT is currently the preferred imaging modality for diagnosing AORCA because of its higher spatial resolution. In coronary angiography, assessing the slit-like orifice and abnormal angulation is challenging, making it difficult to engage the ostium effectively. Furthermore, the three-dimensional structure is often represented in two dimensions, complicating the evaluation [11].

The treatment approach for AORCA varies between symptomatic and asymptomatic patients. Symptomatic patients and those with high-risk features on coronary imaging should be referred for surgical evaluation. In contrast, asymptomatic patients can often be managed conservatively. Surgical intervention in AORCA is always controversial given that patients do not always present with obvious clinical and radiographic evidence of ischemia. According to the guidelines published by the American Association of Thoracic Surgery in 2017, patients with AORCA and symptoms (ischemic chest pain, myocardial infarctions, syncope, ventricular arrhythmias, and history of aborted SCD) should be treated surgically [12]. The surgical options are translocation and reimplantation of RCA to the aorta, unroofing of the ostium, osteoplasty, and coronary artery bypass grafting. Individuals with AORCA who are symptomatic can be treated conservatively and can participate in competitive sports if they have a negative stress test of myocardial perfusion scan and additional normal echocardiography [12]. The treatment strategy for asymptomatic patients is highly controversial as asymptomatic cases with AORCA have been reported. The mortality rate after the surgical repair in patients with AORCA is very low; a latest survey from the Congenital Heart Surgeons Society revealed that only two deaths have been reported out of 113 individuals with AORCA who underwent surgical repair.

Therefore, once AORCA is recognized, careful counseling of the symptomatic and asymptomatic patients should be done regarding surgical options and participation in competitive sports. Our patient had recurrent chest pain and dizziness and underwent coronary artery IMA-RCA bypass grafting. No postoperative complications have been reported so far.

## Conclusions

The anomalous origin of RCA with high interarterial course is a rare congenital coronary anomaly, and individuals can present with myocardial infarction, arrhythmias, syncope, and SCD without atherosclerosis, especially during physical activity. MDCT scan along with stress myocardial perfusion scan is a choice of modality for accurate diagnosis and anatomical assessment for surgical intervention. Surgical referral is recommended in symptomatic patients, and asymptomatic patients can be treated conservatively. Asymptomatic individuals are the greatest challenge to clinicians and cardiologists, and routine testing with ECG and echocardiography is not a sensitive tool to diagnose congenital coronary anomaly; therefore, further investigation is needed.
